# Functional link between sarcoidosis-associated gene variants and quantitative levels of bronchoalveolar lavage fluid cell types

**DOI:** 10.3389/fmed.2023.1061654

**Published:** 2023-02-07

**Authors:** Muntasir Abo Al Hayja, Susanna Kullberg, Anders Eklund, Leonid Padyukov, Johan Grunewald, Natalia V. Rivera

**Affiliations:** ^1^Division of Respiratory Medicine, Department of Medicine Solna, Karolinska Institutet, Karolinska University Hospital, Stockholm, Sweden; ^2^Division of Rheumatology, Department of Medicine Solna, Karolinska Institutet, Karolinska University Hospital, Stockholm, Sweden; ^3^Center of Molecular Medicine (CMM), Karolinska Institutet, Stockholm, Sweden

**Keywords:** sarcoidosis, single nucleotide polymorphisms, immunogenetics, eQTLs, gene regulation, HLA, major histocompatibility complex, broncoalveolar lavage

## Abstract

**Background:**

Sarcoidosis is an inflammatory disease that affects multiple organs. Cell analysis from bronchoalveolar lavage fluid (BALF) is a valuable tool in the diagnostic workup and differential diagnosis of sarcoidosis. Besides the expansion of lymphocyte expression-specific receptor segments (Vα2.3 and Vβ22) in some patients with certain HLA types, the relation between sarcoidosis susceptibility and BAL cell populations’ quantitative levels is not well-understood.

**Methods:**

Quantitative levels defined by cell concentrations of BAL cells and CD4^+^/CD8^+^ ratio were evaluated together with genetic variants associated with sarcoidosis in 692 patients with extensive clinical data. Genetic variants associated with clinical phenotypes, Löfgren’s syndrome (LS) and non-Löfgren’s syndrome (non-LS), were examined separately. An association test *via* linear regression using an additive model adjusted for sex, age, and correlated cell type was applied. To infer the biological function of genetic associations, enrichment analysis of expression quantitative trait (eQTLs) across publicly available eQTL databases was conducted.

**Results:**

Multiple genetic variants associated with sarcoidosis were significantly associated with quantitative levels of BAL cells. Specifically, LS genetic variants, mainly from the HLA locus, were associated with quantitative levels of BAL macrophages, lymphocytes, CD3^+^ cells, CD4^+^ cells, CD8^+^ cells, CD4^+^/CD8^+^ ratio, neutrophils, basophils, and eosinophils. Non-LS genetic variants were associated with quantitative levels of BAL macrophages, CD8^+^ cells, basophils, and eosinophils. eQTL enrichment revealed an influence of sarcoidosis-associated SNPs and regulation of gene expression in the lung, blood, and immune cells.

**Conclusion:**

Genetic variants associated with sarcoidosis are likely to modulate quantitative levels of BAL cell types and may regulate gene expression in immune cell populations. Thus, the role of sarcoidosis-associated gene-variants may be to influence cellular phenotypes underlying the disease immunopathology.

## 1. Introduction

Sarcoidosis is a systemic inflammatory disease of unknown etiology. It is postulated that its cause might result from the interplay between genetic and environmental factors that contribute to disease pathogenesis and the formation of noncaseating granulomas (1).

Sarcoidosis is characterized by at least two distinct clinical phenotypes, Löfgren’s syndrome (LS) and non-Löfgren’s syndrome (non-LS) (2, 3). LS is characterized by acute onset, erythema nodosum and/or bilateral periarticular ankle swelling, and enlarged hilar lymph nodes with a high likelihood of spontaneous remission (4). Patients with non-LS, on the other hand, often present with a more gradual, insidious onset and a heterogeneous disease course (3).

Bronchoalveolar lavage (BAL) fluid has a valuable role in the diagnosis of sarcoidosis. BAL cells comprise several cell subtypes, including macrophages, lymphocytes, and neutrophils, as well as small quantities of basophils and eosinophils (5).

In the appropriate clinical and radiographic findings that are compatible with sarcoidosis, an increased total BAL cell counts, CD4^+^ T cells, and CD4^+^/CD8^+^ ratio (> 3.5) strongly supports the diagnosis of the disease (6).

Recent literature suggests sarcoidosis may be an autoimmune disease (7–9). Immunological studies revealed various types of immune cells implicated in sarcoidosis’s pathogenesis. For instance, it is suggested that a persistent T-helper 1 (Th1) immune response is mediated by antigen-presenting cells (APC), resulting in granuloma formation (10, 11). The role of macrophages and CD4^+^ T cell subtypes has been shown in the immunological profiling of sarcoidosis. Additionally, low levels of IFN-gamma and higher levels of T-bet and ROR-gamma T (12–15) have been reported in LS and non-LS. Nevertheless, a complete understanding of the implication of immune cell populations in the disease is missing.

Genetic investigations have suggested that the genetic architecture of sarcoidosis shares similarities with other autoimmune disorders (7, 16). In particular, the Human Leukocyte Antigen (HLA) region polymorphism plays a significant role in sarcoidosis, especially the *HLA-DRB1* gene polymorphism. Moreover, recent evidence in the field showed that non-HLA gene polymorphism (as reviewed in (17)) also plays a significant role in defining sarcoidosis susceptibility. Our group has revealed that sarcoidosis clinical phenotypes LS and non-LS showed different genetic architectures but shared genomic loci in the major histocompatibility complex (MHC) class II region (18).

Although the biological function of genetic variants associated with sarcoidosis is yet to be elucidated, studies on gene expression suggest that genetic variants associated with the disease play a significant role in gene expression signatures *via* expression quantitative trait loci (eQTLs) (19). Studies on eQTL mapping showed that genetic variation could modify gene expression *via* cis-eQTL regulatory variants, resulting in specific transcriptomic signatures (19, 20). Genetic variants associated with the disease often act as regulatory switches that regulate gene expression in specific compartments leading to the diversification of disease phenotypes. It is postulated that cis-eQTL genetic variants explain a large proportion of the so-called missing heritability (21).

Since immune and inflammatory functions related to sarcoidosis development are presented by multiple immune cells, it is essential to evaluate regulatory mechanisms for controlling cell representation in relevant tissues. BALF is an essential proxy for the representation of immune cells in the lung and is often used in diagnostic procedures for sarcoidosis. The influence of sarcoidosis-associated genetic variants on quantitative levels of BAL cells has not been explored. Therefore, in the present study, we sought to investigate the relationship between sarcoidosis-associated genetic variants and quantitative levels of BALF cell populations in sarcoidosis clinical phenotypes LS and non-LS.

## 2. Materials and methods

### 2.1. Study design and participants

In this study, all included sarcoidosis patients were referred to the Department of Respiratory Medicine, Karolinska University Hospital, Stockholm, Sweden, for an investigational workup of clinically suspected pulmonary sarcoidosis (Table 1). Sarcoidosis diagnosis was based on clinical and radiographical presentation, the absence of an alternative diagnosis, and following the World Association of Sarcoidosis and other Granulomatous Disorders (WASOG) recommendations (22). BAL was performed at the time of the diagnosis. Scadding staging system and spirometry, including diffusion (DLCO), were conducted in most patients. Peripheral blood (PB) sampling for HLA-typing was performed for all patients. Patients signed informed consent, and the study was approved by the Regional Ethical Review Board (Stockholm, Sweden) according to the Declaration of Helsinki.

**Table 1:** Clinical and laboratory characteristics of studied Löfgren’s syndrome (LS) and non-Löfgren’s syndrome (non-LS) (*n* = 692).

	LS (*n* = 288)	Non-LS (*n* = 404)	*p*-Value
Sex (male/female)	160/128	252/152	
Age (median ± SD) years	37 ± 10.47	44 ± 12.38	1.03E-12
**Chest X-ray by Scadding staging system**			
I/II/III/IV	200/88/−/−	125/185/78/16	
**Pulmonary function (median ± sd)**			
VC	95 ± 13.82	90 ± 16.38	9.71E-06
FVC	97 ± 13.35	90 ± 17.00	3.30E-05
FEV1 (l/s)	94 ± 14.24	86 ± 18.46	4.68E-08
TLC	96 ± 25.39	89 ± 28.19	4.27E-06
DLCO	88 ± 22.96	79 ± 30.18	4.95E-09
**BAL analysis (median ± SD)**			
Total number of cells (x10^6^)	25.45 ± 25.38	25.8 ± 20.20	0.7364
Cell concentration (x10^6^/L)	159.15 ± 125.47	172.5 ± 149.18	0.1453
Total number of lymphocytes by FACS	16,433 ± 76,066	21,242 ± 78,140	0.2331
Total number of lymphocytes (%) by FACS	28 ± 16.65	26.8 ± 14.86	0.3721
**BAL cell differential counts (median ± sd)**			
Macrophages (× 10^6^/L)	113.85 ± 98.64	119.57 ± 95.87	0.3503
Macrophages (%)	77 ± 15.32	75.40 ± 16.59	6.24E-02
Lymphocytes (× 10^6^/L)	28.90 ± 51.89	32.55 ± 83.29	4.96E-02
Lymphocytes (%)	20 ± 15.09	21.50 ± 15.91	9.84E-02
Total number of CD3^+^	9,644 ± 9,825	9,267 ± 20,017	0.8483
CD3^+^ (%)	76.45 ± 26.19	64.70 ± 27.81	0.1015
Total number of CD4^+^	7,499 ± 8633.73	5,556 ± 16604.19	0.592
CD4^+^ (%)	83.70 ± 13.64	75.75 ± 15.62	4.75E-03
Total number of CD8^+^	1,057 ± 2879.18	1,651 ± 4150.75	4.67E-02
CD8^+^ (%)	11.40 ± 12.88	18.65 ± 13.81	1.53E-03
CD4^+^/CD8^+^ ratio	7.10 ± 7.20	4.2 ± 5.12	6.51E-08
Neutrophils (x10^6^/L)	1.82 ± 3.42	1.73 ± 35.99	0.5788
Neutrophils (%)	1.10 ± 2.02	1.00 ± 6.71	0.1743
Eosinophils (× 10^6^/L)	0.32 ± 1.39	0.46 ± 4.62	7.65E-02
Eosinophils (%)	0.20 ± 0.62	0.30 ± 1.19	0.06391
Basophils (× 10^6^/L)	0.00 ± 0.35	0.0 ± 1.12	1.486E-02
Basophils (%)	0.00 ± 0.16	0.3 ± 1.17	1.32E-02
*HLA-DRB1* type			
HLA-DRB1*03 positive	196	69	2.20E-16
HLA-DRB1*03 negative	92	335	

### 2.2. Differential BAL cells data

As previously described, a bronchoalveolar lavage procedure was performed in all patients as part of the diagnosis protocol (5). Briefly, a flexible fiber-optic bronchoscope was passed trans-nasally or trans-orally after light sedation and local anesthesia. BAL was performed using sterile phosphate-buffered saline (PBS) solution at 37°C was instilled sequentially. Each aliquot was gradually sucked and collected in a siliconized plastic vessel kept on ice at 4°C. The BAL fluid was gently passed through a Dacron gauze (Millipore, Cork, Ireland) and centrifuged at 400 *g* for 10 min at 4°C. The pellet was resuspended in PBS. The cells were counted in a Bürker chamber. Cytospins of BAL cells were stained with May-Grünwald Giemsa, and differential cell counts were performed (5). Briefly, staining of centrifugal smears was performed according to May-Grunewald Giemsa. Smears were then fixed in methanol for (5 min), 18 ml Methanol, and 33 ml MAY-Grunewald (12 min). Smears were then rinsed with water 2.5 ml Giemsa solution and 48 ml Phosphate buffer 60 mM PH 6.8 (20 min). Smears were then rinsed with water and let dry. May-Grunewald (Mg-500) Sigma-Aldrich and Giemsa (GS500) Sigma-Aldrich were used. BALF cell analysis was defined by the percentage of cells and quantitative levels based on cell concentrations (× 10^6^/L). BALF analysis was performed for macrophages, lymphocytes (CD3^+^, CD4^+^, and CD8^+^ cells), neutrophils, basophils, and eosinophils. The CD4^+^/CD8^+^ ratio was calculated for diagnostic purposes and included as a variable in the study.

### 2.3. Cohort description and BAL cell populations

A Swedish cohort of 692 sarcoidosis cases (288 LS and 404 non-LS) with BALF cell data was examined. Comparisons of distributions of BAL cell concentrations (× 10^6^/L) for macrophages, lymphocytes, CD3^+^, CD4^+^, CD8^+^ cells, neutrophils, basophils, and eosinophils were conducted using the Wilcoxon Rank Sum test function in R software. The same test was applied for the CD4^+^/CD8^+^ ratio. Cohort characteristics are in Table 1.

### 2.4. Statistical analysis

Single nucleotide polymorphisms (SNPs) associated with LS (*n* = 1,933) and non-LS (*n* = 173) published in Rivera et al. (18) were assessed. SNPs were extracted from the ImmunoChip SNP array.

The distribution of BAL cell concentrations (× 10^6^/L) was assessed using the ‘ggqplot’ function in the ggplot2 R package and two normality tests Shapiro–Wilk normality test function (23) and the Anderson-Darling normality test in the nortest R package was applied as secondary assessment. Normalization of BAL cell concentration levels (× 10^6^/L) was applied using a log10-transform.

Outlier detection was conducted using the Grubb test function in the outliers R package. Outliers with a value of *p* of ≤ 0.05 were removed from the dataset. After removing outliers, normality tests were applied for a final assessment.

Association was assessed on normally distributed log10-transformed BAL cell populations and CD4^+^/CD8^+^ ratio in the LS and non-LS groups using an additive model and multivariate linear regression model. The regression coefficient defined by the parameter Beta (or genetic effect) signifies the degree of change in the outcome per unit of change in the quantitative level of the cell type tested. The standard error (SE) signifies the standard deviation of the error in the regression model. Association analysis was performed using PLINK version 1.9 beta (24). The log10-transformed BAL cell subtype was regressed on sex, age, and correlated BAL cell types.

Correlation matrix analysis was conducted using Spearman’s rank correlation method and applied to log10-transformed BAL cell differential counts using the ‘rcorr’ function as implemented in the Hmisc R package. The correlation matrix was visualized as a correlogram using the ‘corrplot’ function in the corrplot R package.

A nominal value of *p* of < 0.05 and a false discovery rate (FDR) < 0.05 (to account for multiple testing) were considered significant. FDR was calculated using the ‘p.adjust’ function in the R package. To account for high linkage disequilibrium (LD; *r*^2^ ≥ 0.8) among associated SNPs, LD pruning was conducted using PriorityPruner version 0.01.4 software^1^ with default parameters, which identified and prioritized tag-SNPs.

### 2.5. eQTL enrichment analysis

To explore the biology of genetic variants associated with BAL cell concentrations, a gene-based cross-tissue expression quantitative trait (eQTLs) enrichment was conducted using Functional Mapping and Annotation of Genome-Wide Association Studies (FUMA GWAS) (25). FUMA eQTL databases included the BIOS QTL browser (26), DICE (27), eQTLcatalogue^2^, eQTLGen (28), GTExt version 8^3^, and scRNA_eQTLs (29) publicly available sources. The selection of eQTL sets was focused on lung, whole blood, and immune cells eQTLs as relevant to the immunopathology of sarcoidosis.

## 3. Results

### 3.1. Clinical characteristics

Clinical characterization of the cohort, including age, sex, chest X-ray according to Scadding, BAL cell analysis, BAL differential cell counts in percentages and concentration levels, lung function, and *HLA-DRB1* genotypes, was performed in LS and non-LS groups, respectively (Table 1).

Normality assessment on distributions of BAL cell concentrations (× 10^6^/L), log10-transformed BAL cell concentrations, and outlier characterization in LS and non-LS groups are shown in Supplementary Figures S1, S2 in Supplementary Info.

Results from correlation matrix analysis using log10-transformed BAL cells quantitative levels and the Spearman correlation method in LS and non-LS groups showed lymphocytes, macrophages, and neutrophils were correlated (*r*^2^ ≥ 0.90 and *p* < 0.05). Similarly, CD3^+^, CD4^+^, and CD8^+^ cell types were correlated (*r*^2^ ≥ 0.99 and *p* < 0.05). Correlograms are shown in Supplementary Figures S3, S4. Linear regression models (illustrated in Supplementary Figure S5) facilitated the identification of several SNP associations with quantitative levels of BAL cells in LS (Table 2) and non-LS (Table 3).

**Table 2:** Association results of LS BAL cell types (log10-tranformed of quantitative levels).

BAL cell type	SNP	CHR	BP	RefSeq genes	Alleles	A1	BETA	SE	*p*	FDR
Macrophages	rs9268861	6	32,429,894	*17 kb 3′ of HLA-DRA*	A/C	A	0.09233	0.02430	1.88E-04	2.50E-02
	rs3129890	6	32,414,273	*1.4 kb 3′ of HLA-DRA*	G/A	G	0.07362	0.02162	7.89E-04	2.50E-02
	rs9267948	6	32,212,233	*20 kb 5′ of NOTCH4*	G/A	G	−0.06988	0.02084	9.44E-04	2.50E-02
	rs2076537	6	32,317,635	*C6orf10*	A/G	A	0.06182	0.02047	2.83E-03	2.50E-02
	rs2050190	6	32,339,076	*C6orf10*	G/A	G	0.05776	0.02084	6.07E-03	2.50E-02
	rs3130349	6	32,147,696	*RNF5*	A/G	A	0.06720	0.02430	6.19E-03	2.50E-02
	rs9267947	6	32,211,218	*19 kb 5′ of NOTCH4*	G/A	G	0.05673	0.02099	7.42E-03	2.50E-02
	rs7750783	6	32,268,080	*C6orf10*	A/G	A	0.06059	0.02276	8.35E-03	2.50E-02
	rs3131063	6	30,763,756	*51 kb 5′ of IER3*	A/G	A	0.05187	0.01967	8.97E-03	2.50E-02
	rs28361060	6	32,303,848	*C6orf10*	A/G	A	−0.07799	0.02976	9.41E-03	2.50E-02
Lymphocytes	rs6457617	6	32,663,851	*29 kb 5′ of HLA-DQB1*	A/G	A	0.15800	0.04782	1.12E-03	1.91E-02
	rs6932517	6	32,678,182	*31 kb 5′ of HLA-DQA2*	G/C	G	0.15530	0.04793	1.38E-03	1.91E-02
	rs1612904	6	32,669,018	*35 kb 5′ of HLA-DQB1*	C/A	C	−0.13750	0.04570	2.94E-03	2.43E-02
	rs4502931	6	32,380,782	*5.9 kb 5′ of BTNL2*	A/T	A	0.13750	0.04825	4.79E-03	2.43E-02
	rs2395153	6	32,345,595	*5.9 kb 5′ of C6orf10*	C/G	C	0.14590	0.05129	4.86E-03	2.43E-02
	rs9469220	6	32,658,310	*24 kb 5′ of HLA-DQB1*	A/G	A	0.13220	0.04745	5.80E-03	2.74E-02
	rs6936428	6	32,739,174	*7.8 kb 5′ of HLA-DQB2*	T/A	T	0.12350	0.04627	8.21E-03	3.67E-02
	rs1165148	6	25,844,710	*616 bp 3′ of SLC17A3*	A/C	A	−0.11400	0.04465	1.14E-02	4.35E-02
	rs1964995	6	32,449,411	*36 kb 3′ of HLA-DRB5*	G/A	G	0.13210	0.05260	1.28E-02	4.35E-02
	rs9275698	6	32,687,973	*21 kb 5′ of HLA-DQA2*	G/A	G	−0.11710	0.04693	1.34E-02	4.37E-02
CD4^+^	rs1265061	6	31,078,257	*741 bp 3′ of C6orf15*	A/C	A	−0.01842	0.00776	2.08E-02	5.00E-02
	rs1043618	6	31,783,507	*HSPA1A*	G/C	G	−0.01968	0.00861	2.59E-02	5.00E-02
	rs4947350	6	32,767,620	*13 kb 3′ of HLA-DOB*	G/A	G	−0.02175	0.00964	2.77E-02	5.00E-02
	rs3852215	6	32,635,501	*2 kb 3′ of HLA-DQB1*	G/A	G	−0.02479	0.01105	2.87E-02	5.00E-02
	rs2071286	6	32,179,896	*NOTCH4*	A/G	A	0.02347	0.01061	3.08E-02	5.00E-02
	rs3129736	6	32,685,953	*23 kb 5′ of HLA-DQA2*	G/A	G	0.02218	0.01019	3.35E-02	5.00E-02
	rs2517529	6	31,076,978	*2 kb 3′ of C6orf15*	C/G	C	−0.01590	0.00752	3.88E-02	5.00E-02
	rs443198	6	32,190,406	*NOTCH4*	G/A	G	−0.01849	0.00877	3.92E-02	5.00E-02
	rs6929408	6	31,300,649	*HLA-B*	A/G	A	−0.01815	0.00863	3.96E-02	5.00E-02
	rs2858331	6	32,681,277	*28 kb 5′ of HLA-DQA2*	G/A	G	−0.02153	0.01041	4.29E-02	5.00E-02
CD8^+^	rs4947350	6	32,767,620	*13 kb 3′ of HLA-DOB*	G/A	G	−0.08248	0.03037	8.63E-03	4.27E-02
	rs2071286	6	32,179,896	*NOTCH4*	A/G	A	0.08473	0.03392	1.53E-02	4.27E-02
	rs2857009	6	32,019,746	*TNXB*	C/G	C	0.07082	0.02844	1.56E-02	4.27E-02
	rs3852215	6	32,635,501	*2 kb 3′ of HLA-DQB1*	G/A	G	−0.08239	0.03593	2.54E-02	4.27E-02
	rs415929	6	32,189,032	*NOTCH4*	G/A	G	0.07073	0.03104	2.63E-02	4.27E-02
	rs3129882	6	32,409,530	*HLA-DRA*	G/A	G	0.07186	0.03159	2.65E-02	4.27E-02
	rs2269424	6	32,132,233	*PPT2-EGFL8*	A/G	A	0.06642	0.03096	3.60E-02	4.27E-02
	rs1383261	6	32,765,451	*15 kb 3′ of HLA-DOB*	A/G	A	−0.06371	0.02993	3.74E-02	4.27E-02
CD3^+^	rs4947350	6	32,767,620	*13 kb 3′ of HLA-DOB*	G/A	G	0.01792	0.00756	2.09E-02	4.18E-02
	rs3129736	6	32,685,953	*23 kb 5′ of HLA-DQA2*	G/A	G	−0.01837	0.00799	2.50E-02	4.18E-02
	rs2071286	6	32,179,896	*NOTCH4*	A/G	A	−0.01902	0.00835	2.64E-02	4.18E-02
	rs3852215	6	32,635,501	*2 kb 3′ of HLA-DQB1*	G/A	G	0.01931	0.00876	3.13E-02	4.18E-02
	rs1265061	6	31,078,257	*741 bp 3′ of C6orf15*	A/C	A	0.01366	0.00621	3.16E-02	4.18E-02
	rs2857009	6	32,019,746	*TNXB*	C/G	C	−0.01248	0.00589	3.84E-02	4.18E-02
	rs6929408	6	31,300,649	*HLA-A*	A/G	A	0.01434	0.00682	3.97E-02	4.18E-02
	rs1043618	6	31,783,507	*HSPA1A*	G/C	G	0.01448	0.00689	3.99E-02	4.18E-02
	rs2213567	6	32,711,655	*HLA-DQA2*	G/C	G	0.01673	0.00804	4.18E-02	4.18E-02
CD4^+^/CD8^+^	rs2259571	6	31,583,827	*AIF1*	C/A	C	0.11710	0.04368	8.03E-03	2.28E-02
	rs1632854	6	30,975,649	*PBMUCL1*	T/A	T	−0.10440	0.03965	9.20E-03	2.28E-02
	rs2531803	6	28,411,220	*ZSCAN23*	C/A	C	0.09191	0.03691	1.37E-02	2.28E-02
	rs2531804	6	28,411,303	*23 bp 5′ of ZSCAN23*	G/A	G	0.08223	0.03836	3.34E-02	3.69E-02
	rs805262	6	31,628,733	*183 bp 5′ of C6orf47*	A/G	A	0.07868	0.03742	3.69E-02	3.69E-02
Neutrophils	rs3130573	6	31,106,268	*PSORS1C2*	G/A	G	−0.12180	0.04123	3.48E-03	4.91E-02
	rs1265099	6	31,105,413	*PSORS1C2*	G/A	G	−0.11890	0.04031	3.54E-03	4.91E-02
	rs241437	6	32,797,684	*TAP2*	G/A	G	−0.10010	0.03878	1.05E-02	4.91E-02
	rs165255	6	29,989,695	*ZNRD1-AS1*	C/G	C	−0.11110	0.04326	1.09E-02	4.91E-02
	rs2516491	6	31,485,101	*6.2 kb 3′ of MICB*	C/A	C	−0.10140	0.04131	1.49E-02	4.91E-02
	rs1264372	6	30,769,726	*57 kb 5′ of IER3*	A/G	A	−0.11060	0.04549	1.58E-02	4.91E-02
	rs2076537	6	32,317,635	*C6orf10*	A/G	A	−0.10110	0.04241	1.80E-02	4.91E-02
	rs3130782	6	30,914,843	*DPCR1*	A/G	A	−0.11580	0.04947	2.01E-02	4.91E-02
	rs3830076	6	32,096,244	*226 bp 5′ of ATF6B*	A/G	A	−0.18680	0.08067	2.15E-02	4.91E-02
	rs241429	6	32,803,840	*TAP2*	A/G	A	0.09105	0.04001	2.39E-02	4.91E-02
Basophils	rs2248462	6	31,446,796	*6.6 kb 3′ of HCG26*	A/G	A	0.61130	0.19580	0.004971	2.93E-02
	rs6457372	6	31,247,121	*7.3 kb 5′ of HLA-C*	A/G	A	0.39250	0.12680	0.005279	2.93E-02
	rs1046089	6	31,602,967	*PRRC2A*	A/G	A	−0.30830	0.10600	0.008132	2.93E-02
	rs2251396	6	31,364,707	*2.9 kb 5′ of MICA*	A/G	A	−0.40920	0.14460	0.009742	2.93E-02
	rs4573120	6	30,767,947	*56 kb 5′ of IER3*	C/A	C	0.27540	0.10190	0.01297	2.93E-02
	rs2471980	6	31,800,868	*1.8 kb 5′ of C6orf48*	G/C	G	−0.32250	0.11960	0.01316	2.93E-02
	rs2516415	6	31,459,742	*6.1 kb 5′ of MICB*	A/G	A	−0.29740	0.11050	0.01332	2.93E-02
	rs2844479	6	31,572,956	*10 kb 5′ of AIF1*	C/A	C	0.34730	0.13470	0.01715	2.93E-02
	rs2259571	6	31,583,827	*AIF1*	C/A	C	0.34730	0.13470	0.01715	2.93E-02
	rs3130481	6	31,839,756	*SLC44A4*	C/G	C	0.36770	0.15400	0.02601	4.17E-02
Eosinophils	rs3823417	6	31,100,869	*PSORS1C1*	A/G	A	0.21760	0.08061	7.81E-03	4.28E-02
	rs2621326	6	32,783,896	*HLA-DOB*	A/G	A	0.14600	0.05598	1.01E-02	4.28E-02
	rs3094122	6	30,728,360	*16 kb 5′ of IER3*	C/A	C	0.14680	0.05666	1.06E-02	4.28E-02
	rs1612904	6	32,669,018	*35 kb 5′ of HLA-DQB1*	C/A	C	−0.14270	0.05607	1.20E-02	4.28E-02
	rs2071286	6	32,179,896	*NOTCH4*	A/G	A	0.17560	0.07253	1.68E-02	4.28E-02
	rs6932517	6	32,678,182	*31 kb 5′ of HLA-DQA2*	G/C	G	0.14040	0.05921	1.91E-02	4.28E-02
	rs7774434	6	32,657,578	*23 kb 5′ of HLA-DQB1*	G/A	G	0.14330	0.06177	2.18E-02	4.28E-02
	rs3095310	6	31,091,447	*PSORS1C1*	A/G	A	0.14980	0.06489	2.25E-02	4.28E-02
	rs2844697	6	30,932,309	*10 kb 3′ of DPCR1*	A/G	A	0.12850	0.05692	2.55E-02	4.28E-02
	rs3799378	6	26,404,374	*BTN3A1*	G/A	G	−0.12480	0.05573	2.67E-02	4.28E-02

SNP = single nucleotide polymorphism; CHR = Chromosome; Position = SNP location in hg19 reference; RefSeq = NCBI gene reference sequence; Alleles = A1 and A2; A1, coded allele; A2 = non-coded allele; BETA = regression coefficient; SE = standard error; *p* = *p*-value; FDR = false discovery rate.

**Table 3:** Association results of non-LS BAL cell types (log10-transformed of quantitative levels).

BAL cell type	SNP	CHR	BP	RefSeq genes	Alleles	A1	BETA	SE	*p*	FDR
Macrophages	rs3117182	6	32,066,819	*TNXB*	A/T	A	−0.06098	0.02450	1.34E-02	2.56E-02
	rs9267992	6	32,220,397	*29 kb 5′ of NOTCH4*	G/A	G	−0.07332	0.02339	1.89E-03	2.03E-02
	rs9268199	6	32,278,635	*C6orf10*	G/A	G	−0.06676	0.02160	2.18E-03	2.03E-02
	rs2076536	6	32,339,348	*C6orf10*	G/A	G	−0.06018	0.01919	1.88E-03	2.03E-02
	rs6913309	6	32,339,840	*183 bp 5′ of C6orf10*	A/T	A	0.07230	0.02441	3.29E-03	2.12E-02
	rs6930777	6	32,351,566	*11 kb 3′ of BTNL2*	A/G	A	0.07866	0.03670	3.29E-02	3.91E-02
	rs9268473	6	32,355,683	*6.8 kb 3′ of BTNL2*	G/A	G	0.05409	0.02020	7.81E-03	2.24E-02
	rs12529049	6	32,357,715	*4.8 kb 3′ of BTNL2*	A/G	A	0.07736	0.03000	1.04E-02	2.24E-02
	rs3129955	6	32,365,840	*BTNL2*	A/G	A	−0.05430	0.02015	7.46E-03	2.24E-02
	rs2395165	6	32,388,144	*13 kb 5′ of BTNL2*	G/A	G	−0.05525	0.02063	7.80E-03	2.24E-02
CD8^+^	rs9277542	6	33,055,247	*HLA-DPB1*	G/A	G	−0.05107	0.02418	3.77E-02	4.88E-02
	rs1953970	10	82,142,176	*14 kb 3′ of DYDC2*	G/A	G	0.05342	0.02271	2.09E-02	4.19E-02
	rs4934167	10	82,203,506	*11 kb 5′ of TSPAN14*	A/G	A	0.05736	0.02209	1.11E-02	4.19E-02
Basophils	rs2858332	6	32,681,161	*28 kb 5′ of HLA-DQA2*	A/C	A	0.23440	0.06674	8.87E-04	2.75E-02
	rs7754768	6	32,420,179	*7.4 kb 3′ of HLA-DRA*	G/A	G	−0.17560	0.06468	8.80E-03	3.04E-02
	rs2647012	6	32,664,458	*30 kb 5′ of HLA-DQB1*	A/G	A	−0.18770	0.07078	1.04E-02	3.04E-02
	rs34102154	6	32,572,106	*14.4 kb of HLA-DRB1*	A/G	A	−0.19200	0.07584	1.42E-02	3.14E-02
	rs4502931	6	32,380,782	*5.9 kb 5′ of BTNL2*	A/T	A	0.17080	0.08040	3.80E-02	4.49E-02
	rs3104389	6	32,595,097	*10 kb 5′ of HLA-DQA1*	A/C	A	0.16800	0.08065	4.18E-02	4.49E-02
	rs1964995	6	32,449,411	*36 kb 3′ of HLA-DRB5*	G/A	G	0.16810	0.08194	4.49E-02	4.49E-02
Eosinophils	rs2857700	6	31,572,481	*11 kb 5′ of AIF1*	A/G	A	0.16160	0.06228	1.01E-02	4.66E-02
	rs9277357	6	33,049,979	*HLA-DPB1*	G/A	G	0.11390	0.04875	2.04E-02	4.66E-02
	rs3117182	6	32,066,819	*TNXB*	A/T	A	0.14230	0.06295	2.49E-02	4.66E-02
	rs3819717	6	32,804,299	*TAP2*	G/A	G	0.10790	0.04879	2.81E-02	4.66E-02
	rs746868	6	31,540,429	*LTA*	G/C	G	0.09538	0.04511	3.56E-02	4.66E-02
	rs3132946	6	32,190,028	*NOTCH4*	A/G	A	0.12810	0.06070	3.60E-02	4.66E-02
	rs2905722	6	31,449,327	*9.1 kb 3′ of HCG26*	A/G	A	0.12060	0.05931	4.32E-02	4.78E-02

SNP = single nucleotide polymorphism; CHR = Chromosome; Position = SNP location in hg19 reference; RefSeq = NCBI gene reference sequence; Alleles = A1 and A2; A1 = coded allele; A2 = non-coded allele; BETA = regression coefficient; SE = standard error; *p* = *p*-value; FDR = false discovery rate.

### 3.2. Löfgren’s syndrome (LS) group

Log10-transformed BAL macrophages associated with 261 LS-SNPs (denoted by 44 tag-SNPs after LD pruning). The lead SNP was rs9268861 (Beta = 0.09233, SE = 0.0243, *p* = 1.88 × 10^−4^, FDR = 2.5 × 10^−2^) located 17 kb of the 3′ of *HLA-DRA* (complete results see Supplementary Table S1). Eighty-five LS-SNPs (denoted by 27 tag-SNPs) were associated with log10-transformed BAL lymphocytes. The lead SNP was rs6457617 (Beta = 0.158, SE = 0.04782, *p* = 1.12E-03, FDR = 1.91E-02) located 29 kb 5′ of *HLA-DQB1* (complete results, see Supplementary Table S2). Cell subgroups within lymphocytes were also identified. Specifically, 41 LS SNPs (denoted by 13 tag-SNPs) were associated with log10-transformed BAL CD4^+^. The lead SNP was rs1265061 (Beta = −0.01842, SE = 0.00776, *p* = 2.08E-02, FDR = 5.00E-02) located 741 bp 3′ of *C6orf15* (complete results, see Supplementary Table S3). Sixteen LS SNPs (denoted by 10 tag-SNPs) were associated with log10-transformed BAL CD8^+^. The lead SNP was rs4947350 (Beta = −0.01842, SE = 0.00776, p = 2.08E-02, FDR = 5.00E-02) located 13 kb 3′ of *HLA-DOB* (complete results, see Supplementary Table S4). Twenty-seven LS SNPs (denoted by 9 tag-SNPs) were associated with log10-transformed BAL CD3^+^. The lead SNP was rs4947350 (Beta = 0.01792, SE = 0.007555, *p* = 2.09E-02, FDR = 4.18E-02) located 13 kb 3′ of *HLA-DOB* (complete results, Supplementary Table S5). Twenty LS SNPs (denoted by 5 tag-SNPs) were associated with log10-transformed BAL CD4^+^/CD8^+^. The lead SNP was rs2248462 (Beta = 0.1171, SE = 0.04368, *p* = 8.03E-03, FDR = 2.28E-02) located in *AIF1* (complete results, see Supplementary Table S6). Ninety-three LS-SNPs (26 tag-SNPs) were associated with log10-transformed BAL neutrophils. The lead SNP was rs3130573 (Beta = −0.1218, SE = 0.04123, *p* = 3.48E-03, FDR = 4.91E-02) located in *PSORS1C2* (complete results, see Supplementary Table S7). Seventy-seven LS SNPs (denoted by 22 tag-SNPs) were associated with log10-transformed BAL basophils. The lead SNP was rs2248462 (Beta = 0.6113, SE = 0.1958, *p* = 4.97E-03, FDR = 2.93E-02) located 6.6 kb 3′ of *HCG26* (complete results, see Supplementary Table S8). Thirty-seven LS SNPs (denoted by 16 tag-SNPs) were associated with log10-transformed BAL eosinophils. The lead SNP rs3823417 (Beta = 0.2176, SE = 0.08061, *p* = 7.81E-03, FDR = 4.28E-02) located in *PSORS1C1* (complete results, see Supplementary Table S9).

### 3.3. Non-Löfgren’s syndrome (non-LS) group

Sixty-nine non-LS SNPs (18 tag-SNPs) were associated with log10-transformed BAL macrophages. SNP-associated were located on chromosomes 6 and 10 (Table 3).

The lead SNP was rs2076536 (Beta = −0.06018, SE = 0.01919, *p* = 1.88 × 10^−3^, FDR = 2.03 × 10^−2^) located in *C6orf10*, (complete results, see Supplementary Table S10). Six non-LS SNPs (denoted by 3 tag-SNPs) were associated with log10-transformed BAL CD8^+^ cells. The lead SNP was rs4934167 (Beta = 0.05736, SE = 0.02209, *p* = 1.11E-02, FDR = 4.19E-02; complete results, see Supplementary Table S11). Thirty-one non-LS SNPs (7 tag-SNPs) were associated with log10-transformed BAL basophils. The lead SNP was rs2858332 (Beta = 0.2344, SE = 0.06674, *p* = 8.87E-04, FDR = 2.75E-02) located 28 kb 5′ of *HLA-DQA2* (complete results, see Supplementary Table S12). 35 SNPs (7 tag-SNPs) were associated with log10-transformed BAL eosinophils. The lead SNP rs2857700 (Beta = 0.1616, SE = 0.06228, *p* = 1.01E-02, FDR = 4.66E-02) located 11 kb 5′ of *AIF1* (complete results, see Supplementary Table S13). For complete results of associations, see Supplementary Tables S1-S13, available in figshare.^4^

As a secondary analysis, we evaluated the effect of the HLA-DBR1*03 allele on non-LS by excluding individuals with the HLA-DRB1*03 allele, given previous knowledge that carrying this allele can lead to a good prognosis, and re-ran the analysis. The findings from this assessment showed similar results for quantitative levels of macrophages, CD8, basophils, and eosinophils and associations with other cell types, including CD3, CD4, and neutrophils (for complete results, see Supplementary Table S14).

### 3.4. Cross-tissue and immune cell expression quantitative trait loci (eQTL) enrichment

Results from eQTL enrichment for LS and non-LS associated with quantitative levels of BALF cell types are summarized in Table 4. Briefly, 143 LS-SNPs associated with BAL cell types were eQTL SNPs (eSNP). Within this set, 46 tag-SNPs were found. eSNPs were correlated with the expression of various immune cells, peripheral blood, and lung using available resources. The most significant LS tag-SNPs for each BAL cell type coupled with eQTL data are shown in Table 5. Likewise, eQTL enrichment identified 35 non-LS SNPs associated with BALF cell types as eSNPs. Within the 35 eSNPs, eight were defined as tag-SNPs. The top non-LS tag SNPs coupled with eQTL data are shown in Table 6. Complete results of eQTL enrichment for LS and non-LS SNPs associated with quantitative levels of BAL cell types are shown in Supplementary Tables S15, S16, https://doi.org/10.6084/m9.figshare.20485038.

**Table 4:** Summary of eQTL mapping for LS and non-LS SNPs associated with BAL cell types.

		LS-BAL	non-LS-BAL
eQTL database	Tissue/cell	eQTL SNPs	eQTL SNPs
BIOSQTL [26]			
	BIOS_eQTL_geneLevel	1921	271
DICE [27]			
	B_cell_naive	64	21
	Monocyte_classical	48	18
	Monocyte_non_classical	58	24
	NK	32	5
	T_CD4_memory_TREG	44	16
	T_CD4_naive	69	12
	T_CD4_naive_activated	49	6
	T_CD4_naive_TREG	40	5
	T_CD4_TFH	42	4
	T_CD4_TH1	23	1
	T_CD4_TH1_17	54	6
	T_CD4_TH17	59	15
	T_CD4_TH2	48	12
	T_CD8_naive	59	13
	T_CD8_naive_activated	36	11
eQTLcatalogue^*^			
	Alasoo_2018_ge_macrophage_naive	–	13
	BLUEPRINT_ge_monocyte	216	52
	BLUEPRINT_ge_neutrophil	212	36
	BLUEPRINT_ge_T-cell	211	22
	CEDAR_B-cell_CD19	65	53
	CEDAR_monocyte_CD14	83	43
	CEDAR_neutrophil_CD15	86	9
	CEDAR_platelet	61	9
	CEDAR_T-cell_CD4	86	41
	CEDAR_T-cell_CD8	113	43
	Fairfax_2012_B-cell_CD19	161	95
	Fairfax_2014_naive	331	107
	GENCORD_ge_fibroblast	46	8
	GENCORD_ge_LCL	132	130
	GENCORD_ge_T-cell	95	82
	GEUVADIS_ge_LCL	350	166
	Kasela_2017_T-cell_CD4	118	42
	Kasela_2017_T-cell_CD8	68	44
	Lepik_2017_ge_blood	630	111
	Naranbhai_2015_neutrophil_CD16	9	4
	Nedelec_2016_ge_macrophage_naive	101	55
	Quach_2016_ge_monocyte_naive	151	47
	TwinsUK_ge_blood	262	126
eQTLGen [28]			
	eQTLGen_cis_eQTLs	3,158	585
	eQTLGen_trans_eQTLs	338	60
GTExV8^**^			
	Lung	906	273
	Whole_Blood	1,185	230
scRNA_eQTLs [29]			
	PBMC	3	–
	T_CD4	3	–

* https://www.ebi.ac.uk/eqtl/Studies/

** https://www.gtexportal.org/home/datasets

**Table 5:** Top LS SNPs associated with BAL cell types identified as eQTL SNPs.

		BIOSQTL [26]	DICE [27]	eQTLcatalogue^*^	eQTLGen [28]	GTExV8^**^
Tag-SNP	Associated BAL cell	Tissue/cell	Tissue/cell	Tissue/cell	Tissue/cell	Tissue/cell
rs9268861	macrophages, lymphocytes	peripheral blood		BLUEPRINT_ge_monocyte, BLUEPRINT_ge_neutrophil, BLUEPRINT_ge_T-cell, CEDAR_neutrophil_CD15, CEDAR_T-cell_CD8, Fairfax_2012_B-cell_CD19, Fairfax_2014_naive, GEUVADIS_ge_LCL, Kasela_2017_T-cell_CD4, Lepik_2017_ge_blood, Nedelec_2016_ge_macrophage_naive, Quach_2016_ge_monocyte_naive, TwinsUK_ge_blood	eQTLGen_cis_eQTLs	Whole_Blood, Lung
rs6457617	lymphocytes, eosinophils	peripheral blood	B_cell_naive, T_CD4_naive, Monocyte_classical, Monocyte_non_classical, T_CD4_TH17, T_CD4_TH2, T_CD4_memory_TREG	CEDAR_B-cell_CD19, Fairfax_2012_B-cell_CD19, Fairfax_2014_naive, GENCORD_ge_LCL, GENCORD_ge_T-cell, GEUVADIS_ge_LCL, Kasela_2017_T-cell_CD4, Kasela_2017_T-cell_CD8, Lepik_2017_ge_blood, Nedelec_2016_ge_macrophage_naive, Quach_2016_ge_monocyte_naive, TwinsUK_ge_blood	eQTLGen_cis_eQTLs, eQTLGen_trans_eQTLs	Whole_Blood, Lung
rs1265061	CD3^+^, CD4^+^	peripheral blood	T_CD4_memory_TREG	BLUEPRINT_ge_monocyte, BLUEPRINT_ge_neutrophil, BLUEPRINT_ge_T-cell, CEDAR_B-cell_CD19, CEDAR_monocyte_CD14, CEDAR_neutrophil_CD15, CEDAR_T-cell_CD4, CEDAR_T-cell_CD8, GENCORD_ge_fibroblast, GENCORD_ge_LCL, GEUVADIS_ge_LCL, Lepik_2017_ge_blood, TwinsUK_ge_blood	eQTLGen_cis_eQTLs	Whole_Blood, Lung
rs4947350	CD3^+^, CD4^+^, CD8^+^	peripheral blood		BLUEPRINT_ge_neutrophil, BLUEPRINT_ge_T-cell, CEDAR_neutrophil_CD15, CEDAR_T-cell_CD8, Fairfax_2012_B-cell_CD19, GENCORD_ge_fibroblast, Lepik_2017_ge_blood	eQTLGen_cis_eQTLs	Whole_Blood, Lung
rs4947350	CD3^+^, CD4^+^, CD8^+^	peripheral blood		BLUEPRINT_ge_neutrophil, BLUEPRINT_ge_T-cell, CEDAR_neutrophil_CD15, CEDAR_T-cell_CD8, Fairfax_2012_B-cell_CD19, GENCORD_ge_fibroblast, Lepik_2017_ge_blood	eQTLGen_cis_eQTLs	Whole_Blood, Lung

* https://www.ebi.ac.uk/eqtl/Studies/

** https://www.gtexportal.org/home/datasets

**Table 6:** Top non-LS tag-SNPs associated with BAL cell types identified as eQTL SNPs.

		BIOSQTL [26]	DICE [27]	eQTLcatalogue^*^	eQTLGen [28]	GTExV8^**^
Tag-SNP	Associated BAL cell	Tissue/cell	Tissue/cell	Tissue/cell	Tissue/cell	Tissue/cell
rs2858332	basophils	peripheral blood	B_cell_naive, Monocyte_classical, Monocyte_non_classical, T_CD4_TH17, T_CD4_memory_TREG	BLUEPRINT_ge_monocyte, BLUEPRINT_ge_neutrophil, BLUEPRINT_ge_T-cell, CEDAR_B-cell_CD19, CEDAR_neutrophil_CD15, CEDAR_T-cell_CD8, Fairfax_2012_B-cell_CD19, Fairfax_2014_naive, GENCORD_ge_LCL, GENCORD_ge_T-cell, GEUVADIS_ge_LCL, Kasela_2017_T-cell_CD4, Kasela_2017_T-cell_CD8, Lepik_2017_ge_blood, Nedelec_2016_ge_macrophage_naive, Quach_2016_ge_monocyte_naive, TwinsUK_ge_blood	eQTLGen_cis_eQTLs	Whole_Blood, Lung
rs9268473	macrophages	peripheral blood	T_CD8_naive_activated	CEDAR_B-cell_CD19, CEDAR_monocyte_CD14, CEDAR_T-cell_CD4, CEDAR_T-cell_CD8, Fairfax_2012_B-cell_CD19, Fairfax_2014_naive, GENCORD_ge_LCL, GENCORD_ge_T-cell, GEUVADIS_ge_LCL, Kasela_2017_T-cell_CD4, Kasela_2017_T-cell_CD8, Lepik_2017_ge_blood, Nedelec_2016_ge_macrophage_naive, TwinsUK_ge_blood	eQTLGen_cis_eQTLs	Whole_Blood, Lung

* https://www.ebi.ac.uk/eqtl/Studies/

** https://www.gtexportal.org/home/datasets

## 4. Discussion

In the present study, we assessed the relationship between LS- and non-LS-associated genetic variants (defined by SNPs) and quantitative levels of BALF cell populations in individuals with sarcoidosis. We also assessed significant findings with eQTL data across tissue and immune cell types relevant to sarcoidosis. Our results showed that sarcoidosis SNPs were significantly associated with the quantitative levels of BALF cells, suggesting a role of DNA variation on BAL cell phenotypes.

Genetic variants associated with LS (18) were significantly associated with quantitative levels of BALF macrophages, lymphocytes (CD3^+^, CD4^+^, CD8^+^, and CD4^+^/CD8^+^ ratio), neutrophils, basophils, and eosinophils, suggesting a wide range of cellular immuno-phenotypes implicated in the disease. LS-SNPs were primarily located on chromosome 6 from 25,844,710 to 33,032,975 base pairs, highlighting the role of the extended major histocompatibility complex (× MHC) region (30) in LS. The xMHC is a vast region with a complex linkage disequilibrium pattern. The ×MHC has been implicated in autoimmune infections and inflammatory diseases (31).

We also observed significant non-LS SNPs associated with the quantitative levels of BALF macrophages, CD8^+^ cells, basophils, and eosinophils. Non-LS SNPs were located on chromosome 6 from 31,449,327 to 33,073,322 base pairs, highlighting the role of the MHC class II subregion, a well-known and extensively studied locus in various autoimmune diseases. Besides this locus, we also observed non-LS SNPs on chromosome 10 from 82,142,176 to 82,206,348 associated with BALF cell quantitative levels. This locus is nearby the *ANXA11* gene, a well-documented locus associated with sarcoidosis (32). *ANXA11* has also been previously reported as a disease modifier and found to be associated with disease chronicity (33–35). A study by Hofmann et al., showed the downregulation of Annexin A11 upon activating CD8^+^ T cells and CD19^+^ B cells in sarcoidosis (36). A review by Kaneko et al., explained the orchestration of immune cells, such as cytotoxic CD4 and CD8 T cells and B cells and antibodies against Annexin A11 described as potential autoantigen could play key roles in the promotion of inflammation and fibrosis (37). The protein encoded by *ANXA11* is a 56-kD antigen recognized by sera in patients with various autoimmune diseases, as reviewed in (38). Compiling these data, we offer further evidence that biological mechanisms underlying sarcoidosis may be autoimmune-mediated. Moreover, LS and non-LS SNPs are associated with other autoimmune diseases (39). Indeed, our findings showed how DNA variation associated with sarcoidosis risk modifies the expression of quantitative levels in BAL cells, which may have an implication on disease development, such as chronic phenotypes, as we have reported in a previous study (40).

The association between sarcoidosis (LS and non-LS) SNPs and quantitative levels of BAL macrophages is noteworthy. Macrophages are regarded as the main source of TNF-alpha, a primary cytokine for granuloma formation. The association between sarcoidosis SNPs and BAL CD8^+^ cells, particularly in non-LS patients, is an attractive finding, as higher levels of cytotoxic lymphocytes have been reported to be associated with chronic phenotypes (41).

Regarding basophils and eosinophils, there is growing evidence that these cell types may have a functional role in developing autoimmune and inflammatory diseases (42, 43). Eosinophils have strong cytotoxic properties and are modulators of other immune cells, which could apply in sarcoidosis. On the other hand, basophils have been reported to play a role in the pathogenesis of lupus nephritis (44) *via* the activation of autoreactive IgE, thymic stromal lymphopoietin, or Toll-like receptors (TLRs). TLR-mediated mechanisms and SNPs associated with different TLRs have been reported to be associated with disease progression in sarcoidosis (45). Interestingly, basophils have been suggested to be involved in B cell survival, activation, and differentiation (46). As new reports emerge, B cells may be key players in the immunopathogenesis of sarcoidosis (47–49). Thus, further investigations are needed to understand the role of basophils and eosinophils in sarcoidosis.

Cumulative evidence shows that eQTLs are strongly enriched for heritability across complex traits (50). eQTL mapping studies have shown that disease-associated loci revealed by genome-wide association studies are enriched in eQTLs and regulatory genes. However, eQTLs explain only a small proportion of the expression heritability as many of eQTLs may be in linkage-disequilibrium (LD) with disease-associated loci, as described in (51). Umans et al. depict the pearls and pitfalls of eQTLs in disease-associated loci and offer insights for future studies.

In this study, we took advantage of comprehensive eQTL sources, which identified several sarcoidosis-associated SNPs associated with quantitative levels of BAL cell types. Chiefly, sarcoidosis-associated SNPs were highly correlated with gene expression of relevant tissues and cell types, including immune cells, blood, and lung. Thus, suggesting a plausible functional mechanism between sarcoidosis gene-variants and regulation of gene expression in lung, blood, and immune cell types relevant to the disease.

The mapping of bulk and single-cell eQTLs on quantitative levels of BAL cells advances our knowledge previously reported in (18), where we showed that sarcoidosis-associated loci were enriched with cis-eQTLs and regulatory SNPs. Specifically, from bulk RNA of blood, we identified over 3,000 cis-eQTLs in LS and over 500 in non-LS, suggesting that regulation of gene expression is likely a dominant mediator for disease risk. Moreover, from bulk RNA in immune cells, we identified several cis-eQTLs significantly correlated with gene expression of B cells, monocytes, macrophages, neutrophils, natural killer (NK) cells, CD8 T cells, and CD4 T cells (i.e., naïve and memory regulatory T cells (Tregs), CD4 Th1 and Th1.17 cells, and CD4 Th2 cells). We also identified cis-eQTLs in the lung and blood derivates (platelets and PBMCs). Notably, although the specificity of cell type in bulk RNA limits the accuracy of these findings, we believe that besides CD4 Th1 cells, other immune cell types that have been previously neglected in the past, are involved in promoting granuloma formation, as highlighted in recent reviews (52, 53). Using single-cell RNA data, we identified 3 single-cell eQTLs in PBMCs and CD4 T cells in LS. sc-eQTLs allow to capture of dynamic processes in different cell-specific types and estimate the variability in gene expression in each cell (54).

Our work refines the immunobiology of sarcoidosis and suggests new incentives for further investigations using single-cell transcriptomics studies and studies for discovering response eQTLs, which are suitable for investigating immune responses under disease treatment conditions (51). Indeed, our study offers continuing knowledge by providing new evidence on the specificity of gene expression in immune cells and their cell type-specific features. It is also worth noting that most sarcoidosis variants lie in non-coding genomic regions, so disease mechanisms are likely driven by common regulatory variants that modify gene expression of target genes in cells and tissues relevant to the disease (55). Nonetheless, our findings should be interpreted with caution in a comparative study as additional evidence is needed to characterize causal variants and determine gene causality.

### 4.1. Limitations of the study

Although our study provides comprehensive information on the relationship between sarcoidosis susceptibility and quantitative levels of BAL cell types, there are some limitations. First, our study is limited by the small number of genetic variants associated with sarcoidosis (LS and non-LS). It is essential to highlight that these variants are derived from the ImmunoChip SNP-array, a customized array focused on genetic variants associated with immune-mediated disorders. The mapping of this SNP array covers a small fraction of genetic variants in the genome. Second, functional interpretation of sarcoidosis-associated genetic variants on the expression of BAL cells is missing as we do not have BAL transcriptomic data in our cohort. eQTL annotation for BALF is also not present in available sources.

## 5. Conclusion

Our finding indicates that sarcoidosis genetic variants associated with LS and non-LS are significantly associated with quantitative levels of BAL cell types. The identification of sarcoidosis-associated SNPs as regulators of gene expression *via* lung, immune cells, and blood eQTLs offers new insights into the immunobiology of sarcoidosis.

## Data availability statement

Summary statistics for this study can be found in the GWAS catalog https://www.ebi.ac.uk/gwas/ under accession numbers GCST005540 and GCST005543. Further association results on SNPs and eQTL enrichment for LS and non-LS SNPs associated with BAL cell types quantitative levels can be found in Supplementary Tables S1–S13, available in Figshare https://doi.org/10.6084/m9.figshare.20485038.

## Ethics statement

The studies involving human participants were reviewed and approved by Ethical Review Board in Stockholm. The patients/participants provided their written informed consent to participate in this study.

## Author contributions

NR designed and executed data analysis. MA was involved in data analysis and drafted the first version of the manuscript. NR, LP, JG, SK, MA, and AE revised the manuscript and provided feedback. SK, JG, and AE phenotyped patients included in the study. All authors contributed to the article and approved the submitted version.

## Funding

This project is funded by the Swedish Heart-Lung Foundation, awarded to NR (grant no. 20170664, 20200506), SK (grant no. 20200163), and JG (grant no. 20190478); Karolinska Institutet Foundation awarded to NR (grant no. FS-2018:0007); Swedish Research Council awarded to LP (grant no. 2018-02884) and JG (grant no. 2019-01034). MA was supported by grants from the Swedish Heart-Lung Foundation, The Swedish Research Council, King Gustaf V’s, and Queen Victoria’s Freemasons’ Foundation through the regional agreement on medical training and clinical research (ALF) between Stockholm County Council and Karolinska Hospital, and Karolinska Institutet. The computations and genomic data handling were enabled by resources provided by the Swedish National Infrastructure for Computing (SNIC) at the Uppsala Multidisciplinary Center for Advanced Computational Science (UPPMAX), partially funded by the Swedish Research Council through grant agreement no. 2018-05973.

## Conflict of interest

The authors declare that the research was conducted in the absence of any commercial or financial relationships that could be construed as a potential conflict of interest.

## Publisher’s note

All claims expressed in this article are solely those of the authors and do not necessarily represent those of their affiliated organizations, or those of the publisher, the editors and the reviewers. Any product that may be evaluated in this article, or claim that may be made by its manufacturer, is not guaranteed or endorsed by the publisher.

## Footnotes

^1^ http://prioritypruner.sourceforge.net/

^2^ https://www.ebi.ac.uk/eqtl/Studies/

^3^ https://www.gtexportal.org/home/datasets

^4^ https://figshare.com/s/6c9651c5b18fd6a6dfe9
