# Supplementary material for: Functional link between sarcoidosis-associated gene variants and quantitative levels of bronchoalveolar lavage fluid cell types
Source: Front Med (Lausanne). 2023 Feb 7;10:1061654. doi: 10.3389/fmed.2023.1061654 (PMC9941743; doi:10.3389/fmed.2023.1061654)

Supplementary Material

1 Supplementary Figures

Figure S1. Normality assessment of BAL cell concentrations in LS

Figure S2. Normality assessment of BAL cell concentrations in non-LS

Figure S3. Correlogram of BAL cell concentrations in LS

Figure S4. Correlogram of BAL cell concentrations in non-LS

2 Supplementary Tables

Supplementary Tables (Figshare link https://figshare.com/s/6c9651c5b18fd6a6dfe9)

Supplementary Table S1. Association results for LS log10-transformed macrophages quantitative levels

Supplementary Table S2. Association results for LS log10-transformed lymphocytes

Supplementary Table S3. Association results for LS log10-transformed CD4

Supplementary Table S4. Association results for LS log10-transformed CD8 quantitative levels

Supplementary Table S5. Association results for LS log10-transformed CD3 quantitative levels

Supplementary Table S6. Association results for LS log10-transformed CD4/CD8

Supplementary Table S7. Association results for LS log10-transformed neutrophils quantitative levels

Supplementary Table S8. Association results for LS log10-transformed basophils quantitative levels

Supplementary Table S9. Association results for LS log10-transformed eosinophils quantitative levels

Supplementary Table S10. Association results for non-LS log10-transformed macrophages quantitative levels

Supplementary Table S11. Association results for non-LS log10-transformed CD8 quantitative levels

Supplementary Table S12. Association results for non-LS log10-transformed basophils quantitative levels

Supplementary Table S13. Association results for non-LS log10-transformed eosinophils quantitative levels

Supplementary Table S14. Association results for non-LS log10-transformed cells excluding HLA-DRB1-*03 positive indviduals

Supplementary Table S15. LS SNPs associated with BAL cell types identified as eQTL SNPs

Supplementary Table S16. non-LS SNPs associated with BAL cell types identified as eQTL SNPs

Figure S1. Normality assessment of BAL cell concentrations in LS

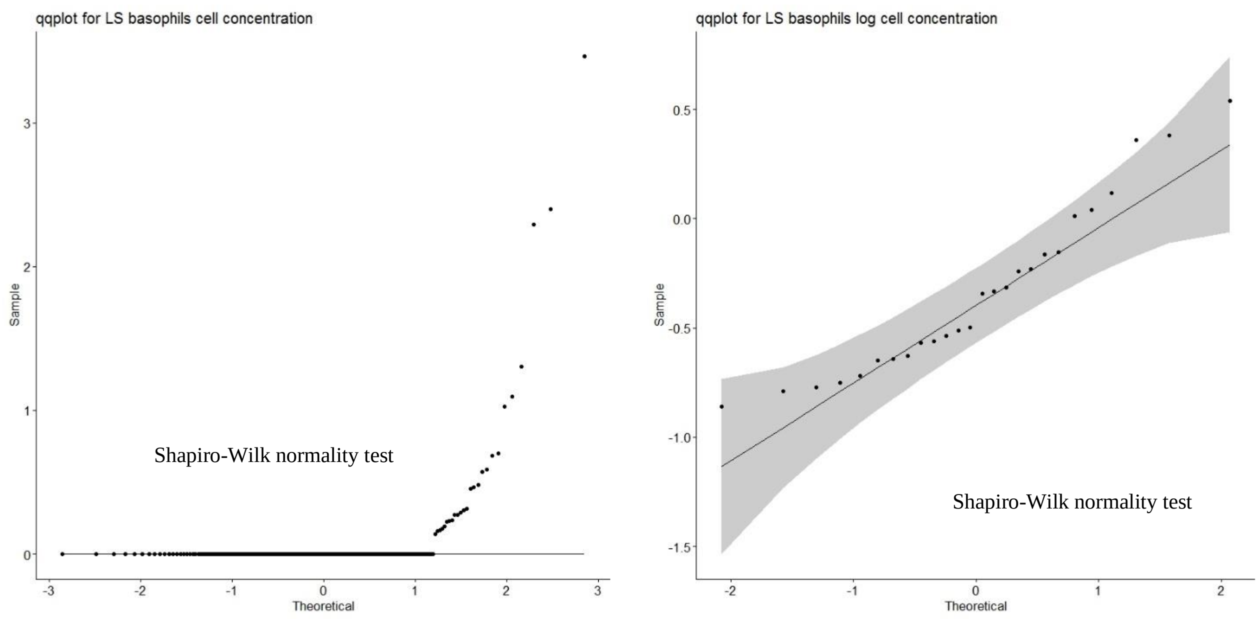

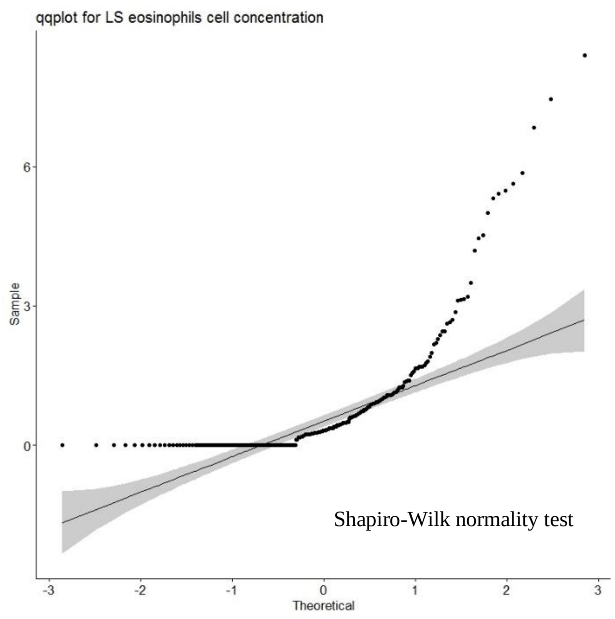
qqplot for LS eosinophils cell concentration
Sample
Theoretical
Shapiro-Wilk normality test

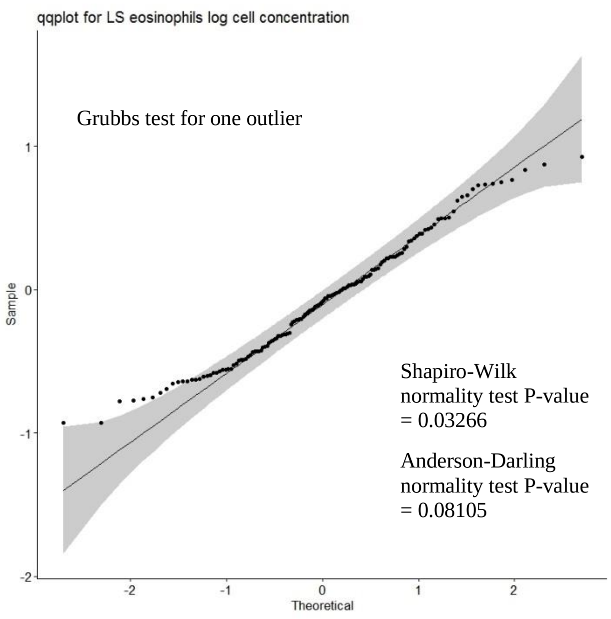
qqplot for LS eosinophils log cell concentration
Grubbs test for one outlier
Sample
Theoretical
Shapiro-Wilk normality test P-value = 0.03266
Anderson-Darling normality test P-value = 0.08105

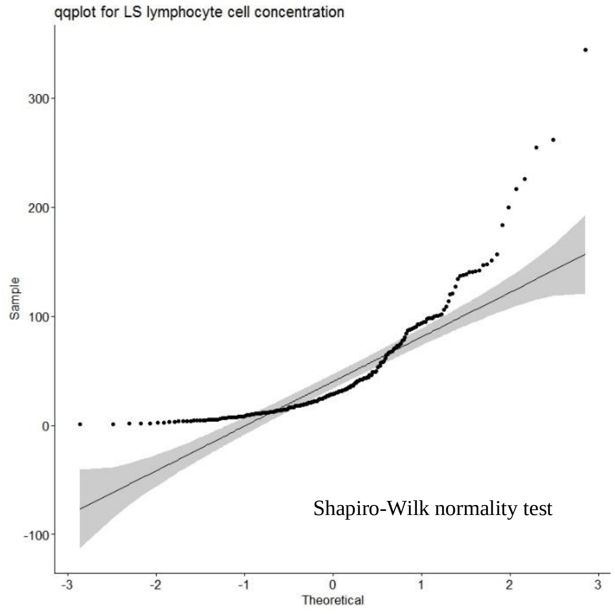
qqplot for LS lymphocyte cell concentration
Sample
Theoretical
Shapiro-Wilk normality test

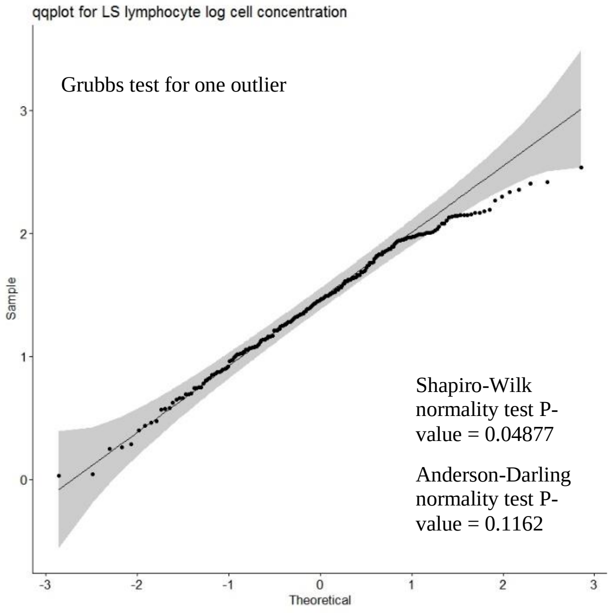
qqplot for LS lymphocyte log cell concentration
Grubbs test for one outlier
Shapiro-Wilk normality test P-value = 0.04877
Anderson-Darling normality test P-value = 0.1162
Sample
Theoretical

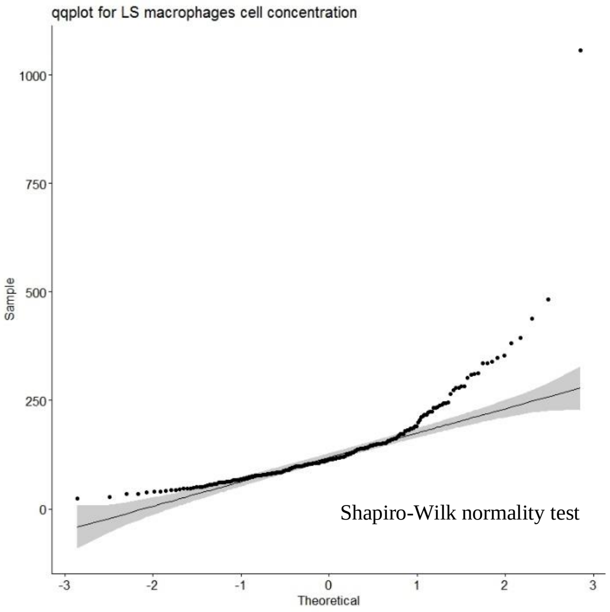
qqplot for LS macrophages cell concentration
Sample
Theoretical
1000
750
500
250
0
-3
-2
-1
0
1
2
3
Shapiro-Wilk normality test

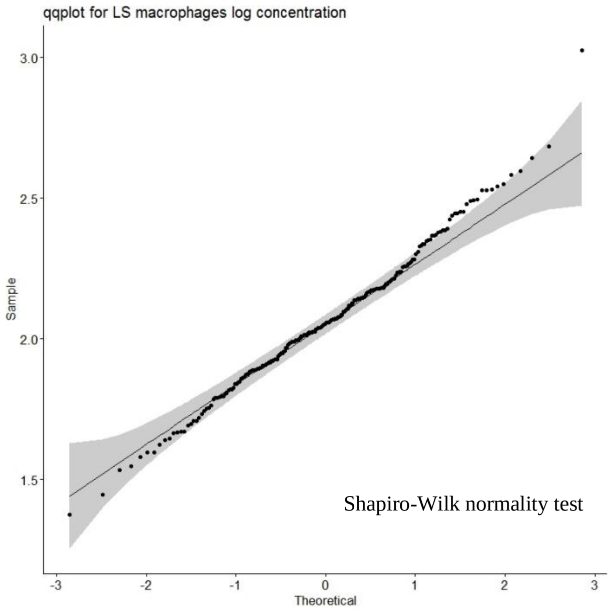
qqplot for LS macrophages log concentration
Sample
Theoretical
3.0
2.5
2.0
1.5
-3
-2
-1
0
1
2
3
Shapiro-Wilk normality test

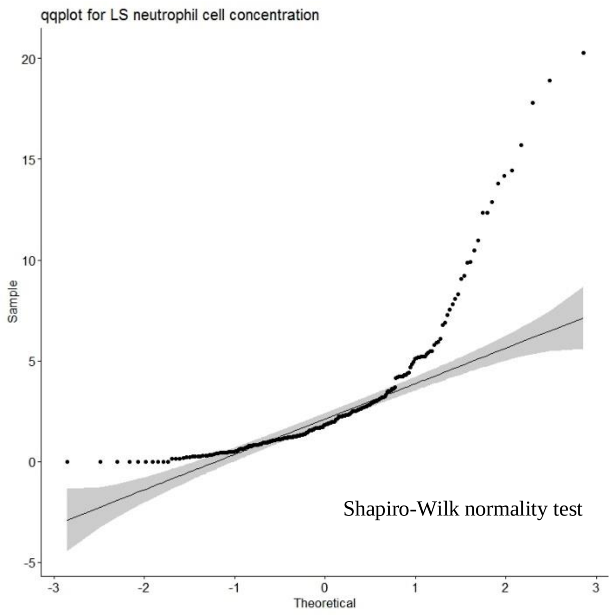
qqplot for LS neutrophil cell concentration
Sample
Theoretical
Shapiro-Wilk normality test

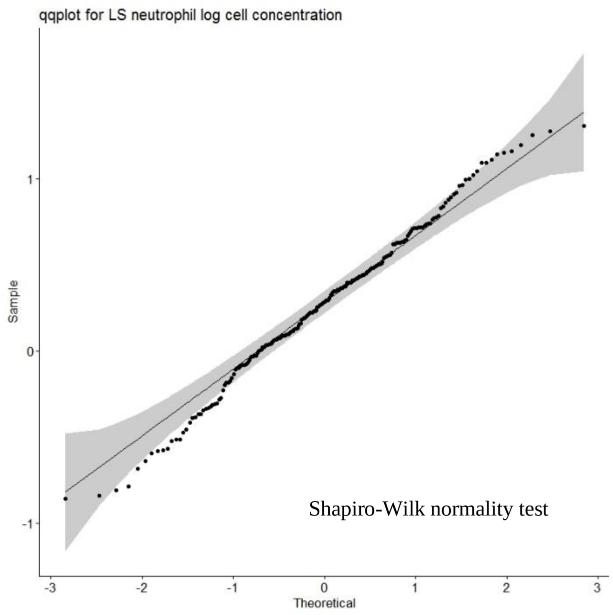
qqplot for LS neutrophil log cell concentration
Sample
Theoretical
Shapiro-Wilk normality test

Figure S2. Normality assessment of BAL cell concentrations in non-LS

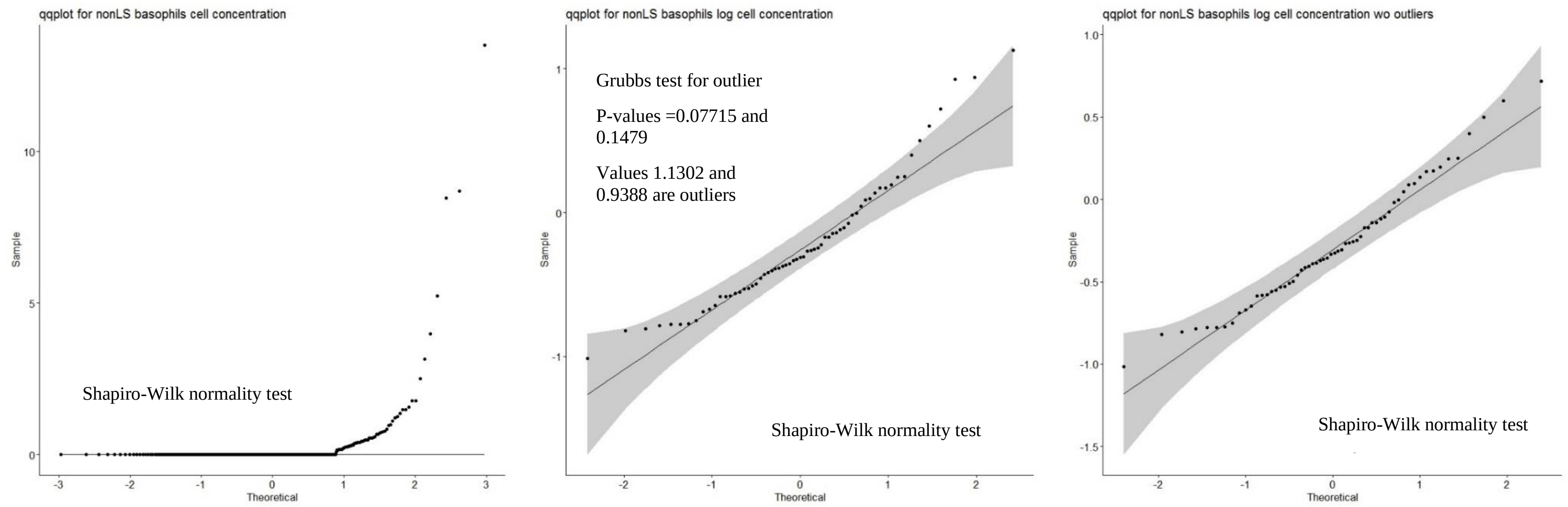

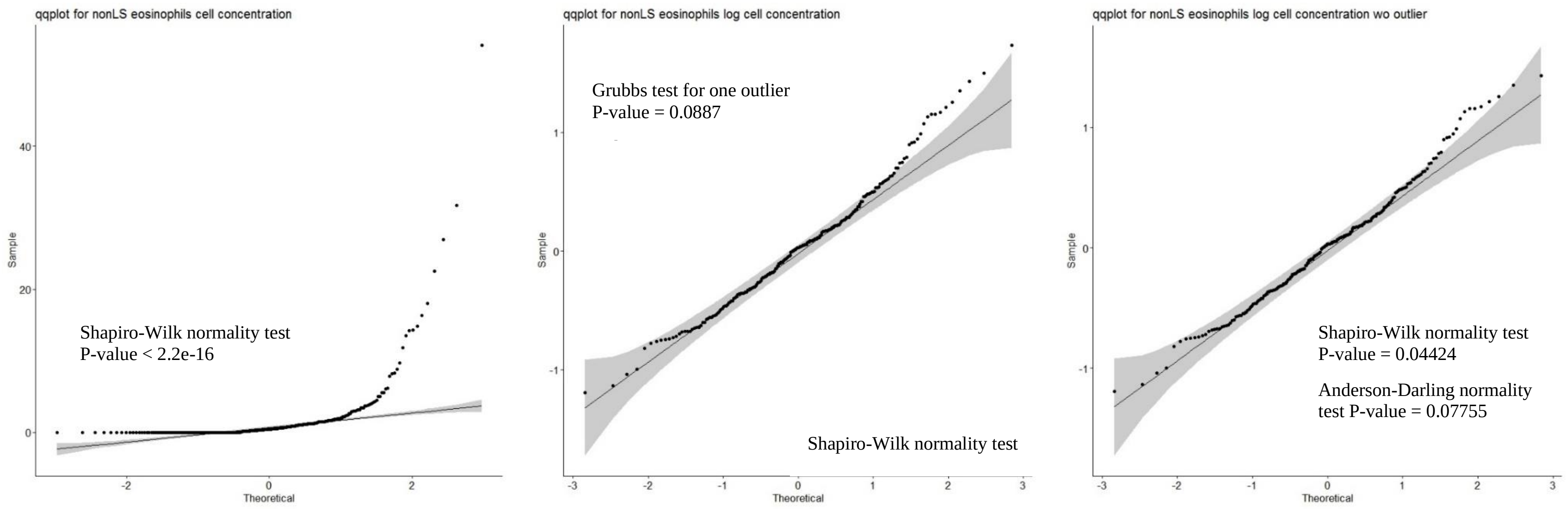
qqplot for nonLS eosinophils cell concentration
Shapiro-Wilk normality test
P-value < 2.2e-16
Sample
Theoretical
qqplot for nonLS eosinophils log cell concentration
Grubbs test for one outlier
P-value = 0.0887
Shapiro-Wilk normality test
Sample
Theoretical
qqplot for nonLS eosinophils log cell concentration wo outlier
Shapiro-Wilk normality test
P-value = 0.04424
Anderson-Darling normality
test P-value = 0.07755
Sample
Theoretical

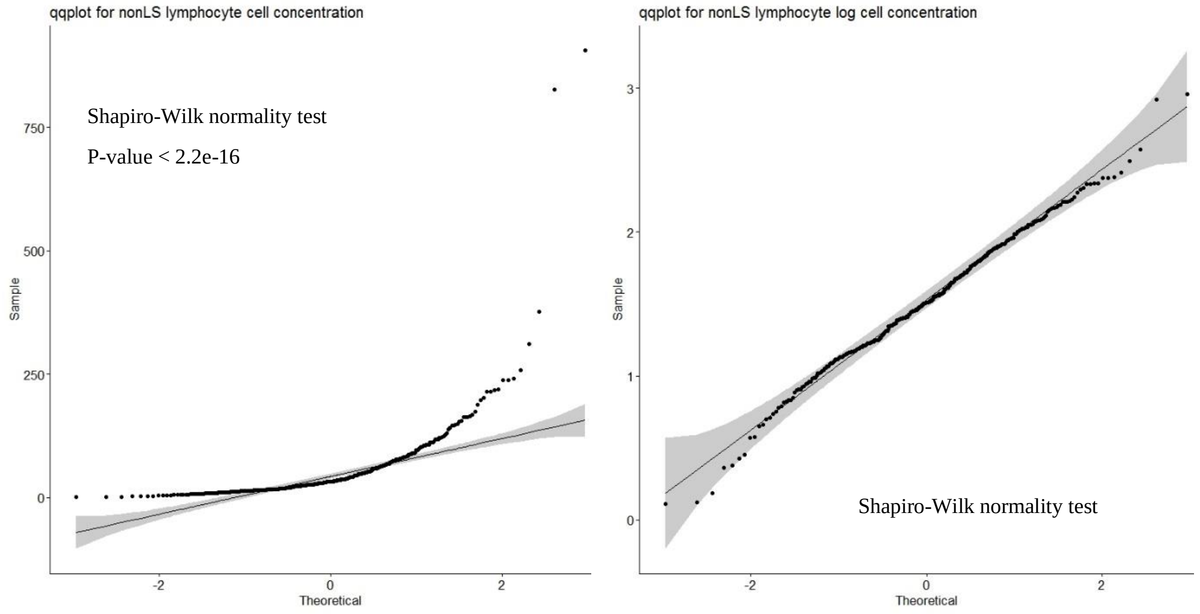
qqplot for nonLS lymphocyte cell concentration
Shapiro-Wilk normality test
P-value < 2.2e-16
Sample
Theoretical
qqplot for nonLS lymphocyte log cell concentration
Shapiro-Wilk normality test
Sample
Theoretical

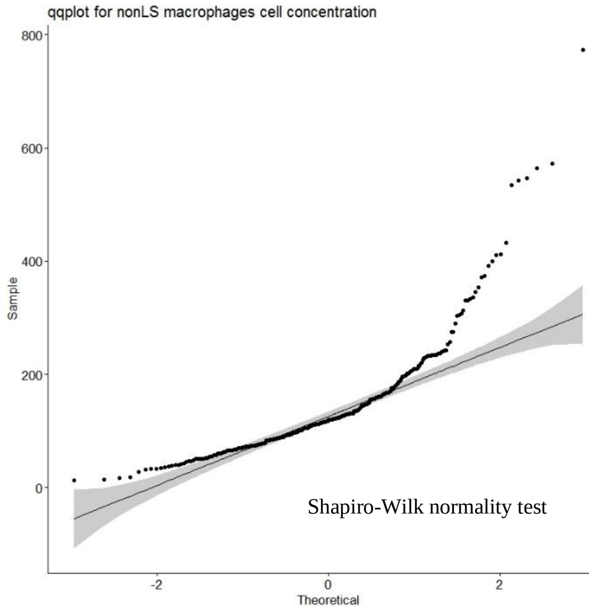
qqplot for nonLS macrophages cell concentration
800
600
400
200
0
Sample
-2
0
2
Theoretical
Shapiro-Wilk normality test

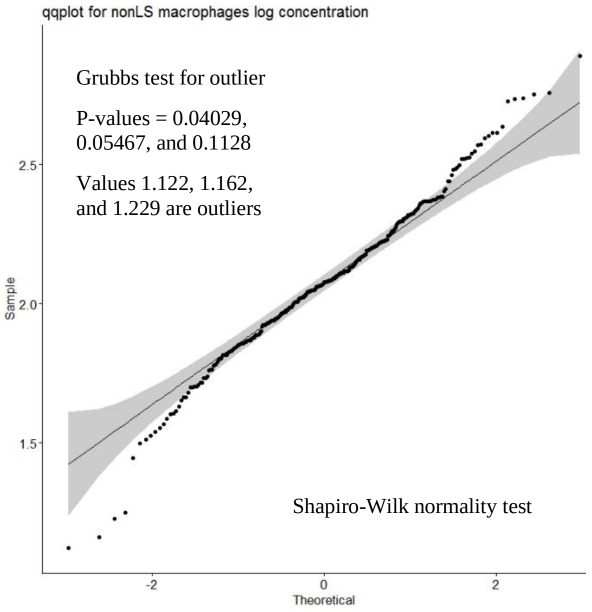
qqplot for nonLS macrophages log concentration
Grubbs test for outlier
P-values = 0.04029, 0.05467, and 0.1128
Values 1.122, 1.162, and 1.229 are outliers
2.5
2.0
1.5
Sample
-2
0
2
Theoretical
Shapiro-Wilk normality test

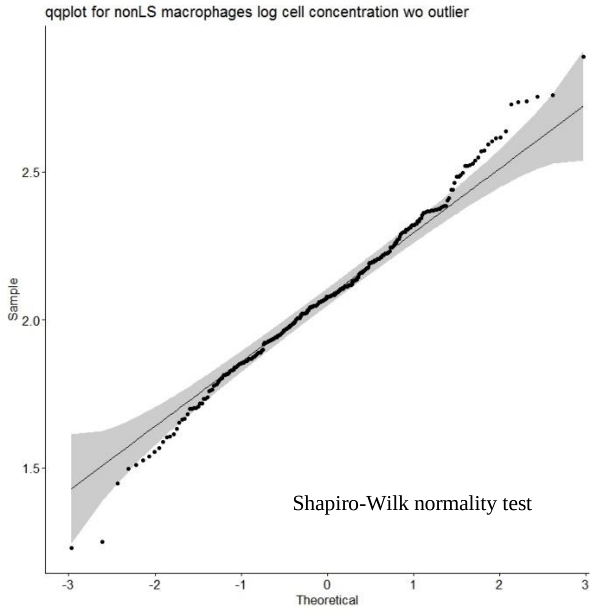
qqplot for nonLS macrophages log cell concentration wo outlier
2.5
2.0
1.5
Sample
-3
-2
-1
0
1
2
3
Theoretical
Shapiro-Wilk normality test

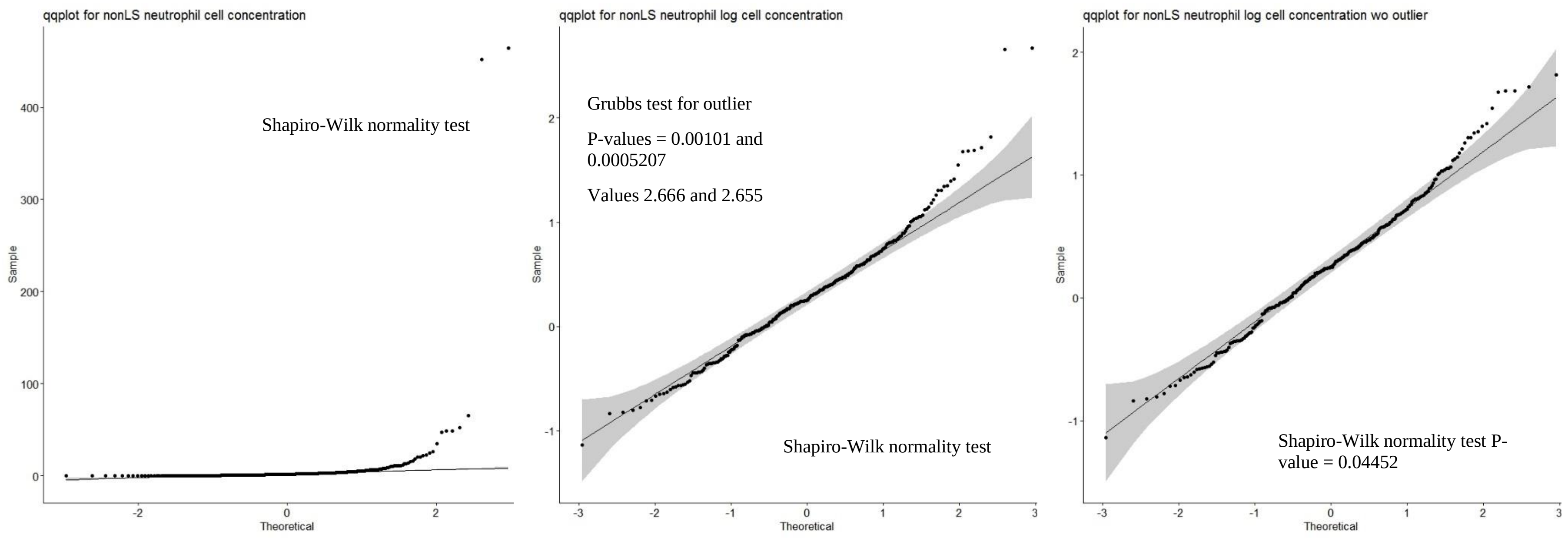
qqplot for nonLS neutrophil cell concentration
Shapiro-Wilk normality test
Sample
Theoretical
qqplot for nonLS neutrophil log cell concentration
Grubbs test for outlier
P-values = 0.00101 and 0.0005207
Values 2.666 and 2.655
Shapiro-Wilk normality test
Sample
Theoretical
qqplot for nonLS neutrophil log cell concentration wo outlier
Shapiro-Wilk normality test P-value = 0.04452
Sample
Theoretical

Figure S3. Correlogram of BAL cells in LS

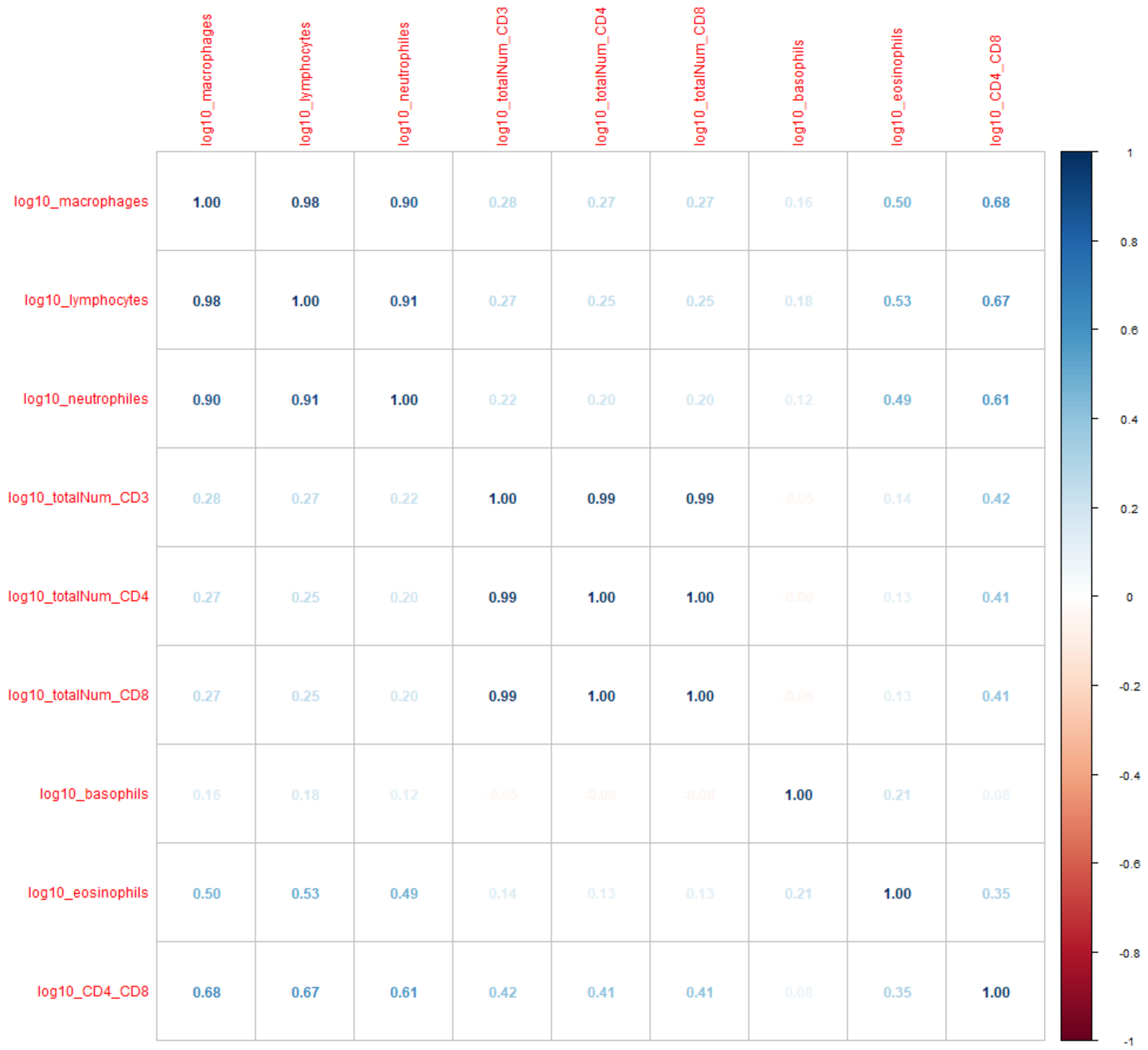

Figure S4. Correlogram of BAL cells in non-LS

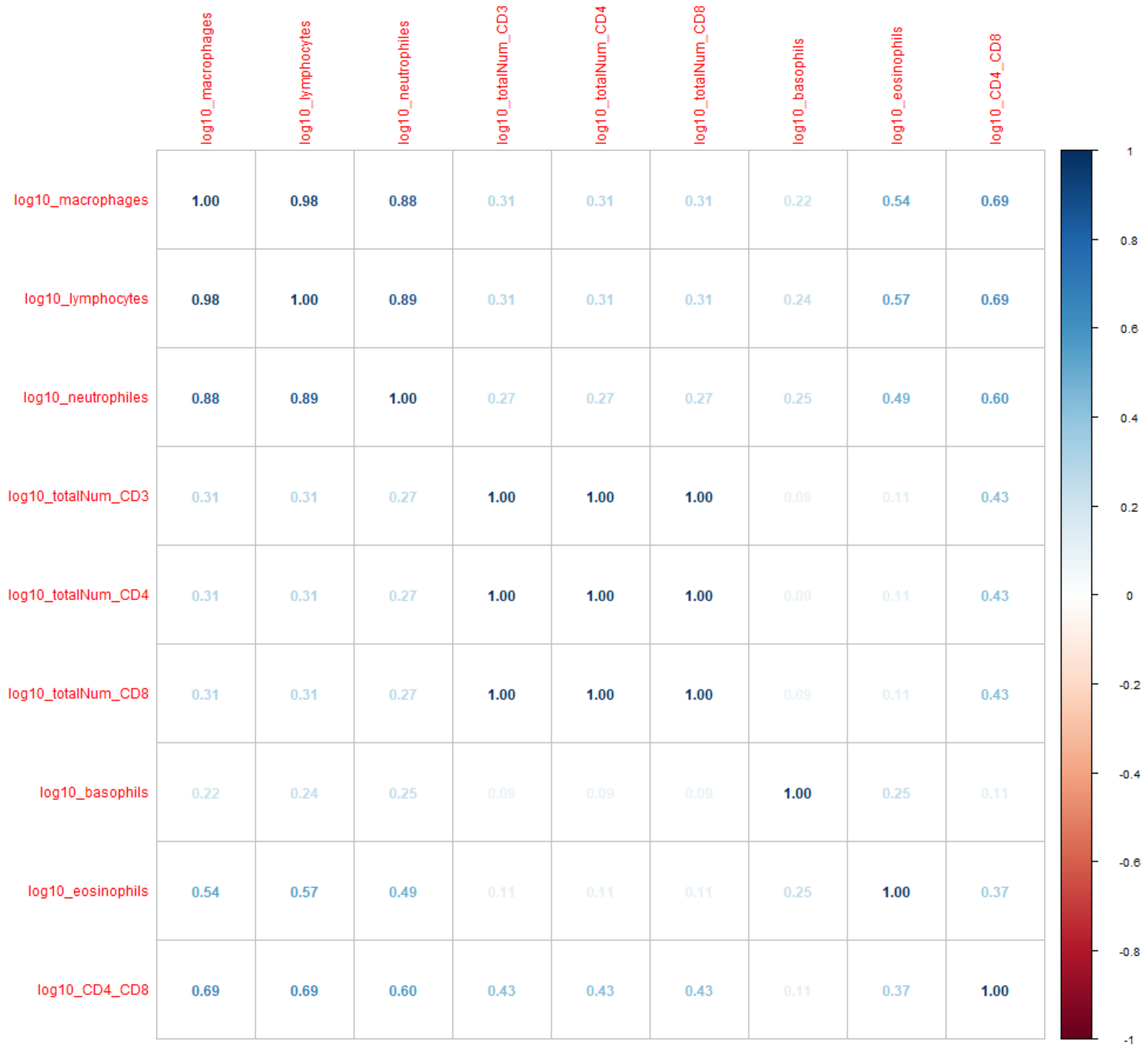

Figure S5. Flowchart illustrating multivariate model

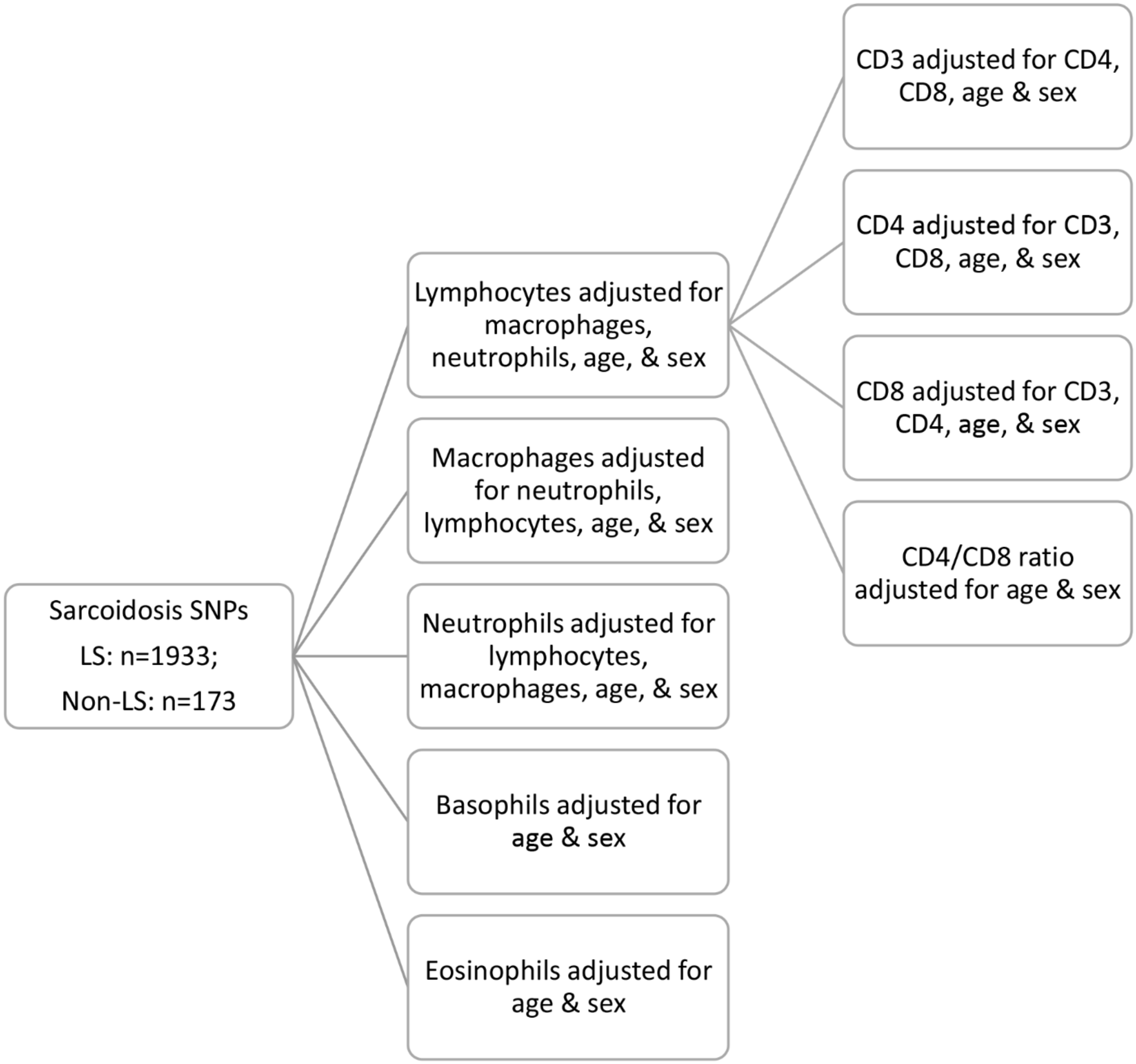

Supplement: Supplementary file 1 [file Data_Sheet_1.pdf]
